# Upper and lower respiratory tract microbiota in horses: bacterial communities associated with health and mild asthma (inflammatory airway disease) and effects of dexamethasone

**DOI:** 10.1186/s12866-017-1092-5

**Published:** 2017-08-23

**Authors:** Stephanie L. Bond, Edouard Timsit, Matthew Workentine, Trevor Alexander, Renaud Léguillette

**Affiliations:** 1 Faculty of Veterinary Medicine, University of Calgary, Calgary, AB Canada; 2 Lethbridge Research Centre, Agriculture and Agri-Food Canada, Lethbridge, AB Canada

**Keywords:** Equine, Metagenomic, 16sRNA, Transtracheal wash, Nasopharyngeal swab, Bronchoalveolar lavage

## Abstract

**Background:**

The microbial composition of the equine respiratory tract, and differences due to mild equine asthma (also called Inflammatory Airway Disease (IAD)) have not been reported. The primary treatment for control of IAD in horses are corticosteroids. The objectives were to characterize the upper and lower respiratory tract microbiota associated with respiratory health and IAD, and to investigate the effects of dexamethasone on these bacterial communities using high throughput sequencing.

**Results:**

The respiratory microbiota of horses was dominated by four major phyla, Proteobacteria (43.85%), Actinobacteria (21.63%), Firmicutes (16.82%), and Bacteroidetes (13.24%). Fifty genera had a relative abundance > 0.1%, with Sphingomonas and Pantoea being the most abundant. The upper and lower respiratory tract microbiota differed in healthy horses, with a decrease in richness in the lower airways, and 2 OTUs that differed in abundance. There was a separation between bacterial communities in the lower respiratory tract of healthy and IAD horses; 6 OTUs in the tracheal community had different abundance with disease status, with *Streptococcus* being increased in IAD horses. Treatment with dexamethasone had an effect on the lower respiratory tract microbiota of both heathy and IAD horses, with 8 OTUs increasing in abundance (including *Streptococcus*) and 1 OTU decreasing*.*

**Conclusions:**

The lower respiratory tract microbiota differed between healthy and IAD horses. Further research on the role of *Streptococcus* in IAD is warranted*.* Dexamethasone treatment affected the lower respiratory tract microbiota, which suggests that control of bacterial overgrowth in IAD horses treated with dexamethasone could be part of the treatment strategy.

**Electronic supplementary material:**

The online version of this article (doi:10.1186/s12866-017-1092-5) contains supplementary material, which is available to authorized users.

## Background

Horses can suffer from airway inflammation, resulting in severe or mild asthma [1, 2]. Severe equine asthma (Recurrent Airway Obstruction) is less common than mild asthma in North America, and manifest by increased respiratory effort at rest [1, 3]. Mild equine asthma (also known as Inflammatory Airway Disease [IAD]) affects up to 66% of the equine population [4] and results in a mild increased resistance to airflow during exercise within the lower airways [5–8]. Clinical signs of IAD are typically subtle at rest, with horses exhibiting normal respiratory effort and occasional coughing; at work, increased nasal discharge, cough and poor performance are observed [8].

Although IAD is primarily an inflammatory process, an infectious component is highly suspected [9–11]. The presence of *Streptococcus zooepidemicus*, *Streptococcus pneumoniae, Actinobacillus* spp., and *Mycoplasma equihinis* in tracheal samples was recently associated with IAD, indicating that composition of the lower respiratory tract microbiota could contribute to the pathogenesis [9]. Furthermore, microbial composition and diversity of the bronchial airways in humans with sub-optimally controlled asthma has been associated with the degree of bronchial hyperresponsiveness, suggesting that lower airway bacterial communities play a role in asthma pathogenesis [12]. However, as there is limited evidence and controversy on associating IAD with bacterial populations in the trachea, further research is required to confirm this association in horses. Furthermore, there is a need to comprehensively describe the bacterial communities present in health and disease as the overall composition of the bacterial communities, rather than the presence of individual species, is important in defining health and disease [12–14].

Airway inflammation associated with IAD is primarily treated with parenteral corticosteroids (i.e. dexamethasone) [8]. Corticosteroids are effective at controlling airway inflammation and inhibiting airway hypersensitivity and hyperreactivity, thus improving pulmonary function [15]. However, as corticosteroids can cause immunosuppression within the respiratory tract [16], treating IAD with dexamethasone could potentially influence the lower respiratory tract bacterial communities, promoting the overgrowth of specific bacteria, which may in turn contribute to recrudescence of disease upon cessation of treatment. Currently, the impact of dexamethasone on the equine respiratory microbiota is unknown.

The objectives of the current study were therefore (i) to characterize the upper and lower respiratory tract microbiota associated with health and mild IAD (mild asthma) and, (ii) to investigate the effects of dexamethasone on these bacterial communities, using high throughput sequencing.

## Methods

### Ethics statement

This study was conducted in strict accordance with the recommendations of the Canadian Council of Animal Care. The research protocol was reviewed and approved by the University of Calgary Veterinary Sciences Animal Care Committee (AC17-0036).

### Animals and study design

**Fig. 1:**
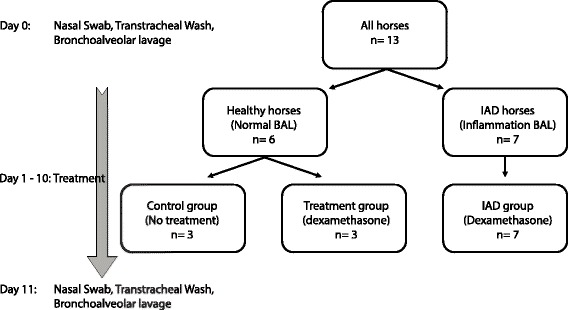
Representation of protocol and treatment group allocation. Horses (*n* = 13) were allocated into two groups on the basis of their bronchoalveolar lavage (BAL); healthy horses (*n* = 6) with a normal BAL, and horses with inflammatory airway disease (IAD, *n* = 7) with an inflammatory BAL

Thirteen deconditioned Thoroughbred horses (geldings) used for chuckwagon racing were studied over a period of 12 days. All horses had a history of coughing, resided on the same property (Okotoks, AB, Canada) and were kept outside in dirt paddocks. They were fed a diet of second-cut alfalfa hay for the duration of the trial, beginning a minimum of 2 days before initial sampling. Nasopharyngeal swabs (NPS), percutaneous transtracheal washes (TTW) and bronchoalveolar lavages (BAL) were performed on all horses (*n* = 13) on day 0 (Fig. 1). Horses were then allocated on day 1 into one of three groups based on their BAL cytology (IAD versus healthy) and random selection (among healthy horses); IAD (horses with mild equine asthma; *n* = 7), DEX (healthy horses treated with dexamethasone; *n* = 3) and CONTROL (healthy horses not treated with dexamethasone; *n* = 3). Horses were considered to have mild equine asthma based on the following inclusion criteria: 1. a BAL with increased percentage of mast cells (> 3%) or/and eosinophils (> 0.5%) or/and neutrophils (> 10%), 2. absence of laboured breathing at rest [8].

Horses in IAD and DEX groups were then administered dexamethasone (20 mg, IM) every morning for 10 days. No other medications were given to horses for the duration of the trial. On day 11, NPS, TTW and BAL procedures were repeated (Fig. 1).

### Sampling procedures

Horses were pre-medicated with acepromazine maleate (0.07-0.08 mg/kg, IM/IV) approx. Thirty minutes prior to procedures. Horses were sedated to effect with xylazine hydrochloride (0.4 – 0.5 mg/kg, IV) and butorphanol tartrate (0.05-0.1 mg/kg). Following sedation, nasopharyngeal swabs (NPS) were performed first, followed by the percutaneous transtracheal washes (TTW) and then by the bronchoalveolar lavages.

Nasopharyngeal swabs (NPS) were collected as described previously [17], using long guarded swabs (27 cm) with a rayon bud (Dryswab Veterinary Laryngeal, Medical Wire and Equipment, Corsham, England). Two NPS were obtained per horse (one per nasal cavity). Control swabs (*n* = 2) were collected each sampling day, with the tip of the swab being exposed to the barn air. Immediately after collection, NPS were placed into an Amies transport media (1.0 mL) and refrigerated at 4 °C. Samples were processed within 10 h of collection. At processing, each rayon tip was removed from the Amies transport media, which was then transferred into a sterile 1.5 mL microfuge tube. The Amies and tip were stored in the microfuge tube and original transport container respectively, at −80 °C pending DNA extraction.

Percutaneous transtracheal washes were performed as described previously [18]. Briefly, a 10cmx15cm area over the mid-cervical trachea was clipped and surgically prepared. After subcutaneous local anaesthesia (2% lidocaine, 5 ml), a stab incision was made through the skin and subcutaneous tissues at the mid-tracheal level and an equine TTW kit was utilized (Mila International, Item number: TW1228) according to manufacturer’s instructions. Sterile saline (15 ml) was injected through the catheter, and aspirated immediately. The aspirate was immediately transferred into a sterile 10 mL plain tube [19] and stored at 4 °C. Controls (*n* = 2) were also obtained using the same sterile saline, which was flushed into the catheter portion of a sterile TTW kit. Samples were processed within 10 h of collection. At processing, particulate matter was re-suspended in aspirate through gentle agitation via pipette and 3.0 mL of aspirate was transferred into 2 sterile 1.5 ml microfuge tubes, which were then centrifuged (14,000 rpm, 3 mins) to pellet the bacteria. The resulting pellet was stored in the microfuge tubes at −80 °C pending DNA extraction.

Bronchoalveolar lavages were performed as described previously [4], with modifications. Briefly, a balloon-tipped BAL tube (Mila International, SKU: BAL300) was inserted into a nostril and progressively passed until its tip was wedged against the wall of a bronchus. During insertion of the BAL tube through the airways, several small boluses of a 0.5% solution (120 mL total/horse) of lidocaine hydrochloride were administered to desensitize the bronchial mucosa. Two boluses (250 ml/bolus) of sterile isotonic saline (0.9% NaCl) solution were sequentially and rapidly instilled in the bronchus and then immediately aspirated by use of sterile 60 ml syringes. Lavage fluid was recovered and transferred into two 10 mL sterile EDTA tubes and kept on ice until analysis, which was performed within 6 h of sample collection. Preparation of slides was performed with 400 μl of BAL fluid, which was centrifuged using a Cytospin (90 X g for 5 min) to spread the pellet on the slide and stained with modified Wright-Giemsa stain. A differential count was performed on a minimum of 400 cells; epithelial cells were not included in the differential count. A paired nonparametric test (Wilcoxon Signed Rank Test) was used to compare BAL variables between day 0 and 12. A *p*-value < 0.05 was considered significant.

### DNA extraction

Total DNA was extracted from NPS and TTW samples using a Qiagen DNEasy Tissue kit (Qiagen Inc., Mississauga, ON, Canada) with the following modifications; briefly, after thawing, the microfuge tubes containing the Amies (NPS samples) and TTW fluid were centrifuged (13,000×g for 5 min) again to ensure that bacteria were pelleted before the DNA extraction procedures. The supernatant was discarded. The rayon tip of the NPS was removed from the applicator and then placed in microfuge tube with bacterial pellet for each sample. The pellets were re-suspended in 180 μl of enzymatic buffer containing mutanolysin (300 U ml^−1^) and lysozyme (20 mg ml^−1^). The mixtures were vortexed and then incubated for 1 hour at 37 °C. Twenty-five μl of proteinase K and 200 μl Buffer AL (without ethanol) were then added, followed by vortexing and incubation at 56 °C for 30 min. Approximately 300 mg of 0.1 mm zircon/ silica beads were added and mixed using a Tissue Lyser II (Qiagen) at 30 Hz for 5 min. The mixtures were then centrifuged (13,000×g for 5 min), and 200 μl of ethanol was added to the supernatants, followed by vortexing. The remainder of the protocol of the DNEasy Tissue Kit was followed as per manufacturer instructions. Extracted DNA was stored at −80 °C until amplification and sequencing. Blank negative controls (kit only) were included in triplicate during DNA extraction.

### Amplification and sequencing

The standard Illumina 16S metagenomics library preparation protocol was used (available at: https://support.illumina.com/content/dam/illumina-support/documents/documentation/chemistry_documentation/16s/16s-metagenomic-library-prep-guide-15044223-b.pdf). All amplification and sequencing steps were carried out at University Core DNA Services, Sequencing and Genetic Analysis Lab (University of Calgary, AB, Canada). The 16S Amplicon PCR forward primer (5’TCGTCGGCAGCGTCAGATGTGTATAAGAGACAGCCTACGGGNGGCWGCAG) and reverse primer (5’GTCTCGTGGGCTCGGAGATGTGTATAAGAGACAGGACTACHVGGGTATCTAATCC) were used to amplify the V3 and V4 regions of the 16S rRNA gene. Illumina sequencing adapters and dual-index barcodes were added to the amplicon target to allow for library pooling prior to sequencing. Briefly, 16S rRNA gene amplicons were generated using a KAPA HiFi HotStart ReadyMix Kit (Kapa Biosystems) with the following PCR conditions: a 3 min initial denaturation at 95 °C followed by 25 cycles of 95 °C for 30 s, 55 °C for 30 s, and 72 °C for 30 s, with a final extension of 5 min at 72 °C. Amplicon were then purified with Agencourt AMPure XP beads (Beckman Coulter Inc., ON, Canada), and sequenced on an Illumina MiSeq system (Illumina Inc., Victoria, BC, Canada) using the 2 × 300 bp paired-end sequencing kit. Negative controls were included in triplicate during amplification and sequencing.

### Operational taxonomic unit (OTU) table construction

Raw reads were processed with cutadapt 1.8.3 [20] to remove the primer sequences and any preceding adaptors. Subsequent processing was done using the UPARSE pipeline [21] as implemented in usearch 8.1.1861. The forward and reverse reads were merged using the fastq_mergepairs option of usearch and subsequently filtered with usearch –fastq_filter and an expected error (EE) cut-off of 1 [22] and truncated to maximum length of 420 bp. The filtered reads were de-replicated using usearch -derep_fulllength and then clustered at 97% identity with usearch -cluster_otus and the option ‘-minsize 2’ to remove singleton reads prior to clustering. Taxonomy was assigned to the representative sequences using the RDP naïve Bayesian classifier [23] as implemented in ‘assign_taxonomy’ function in the R package dada2 [24] using the RDP training set 14. The final OTU table was constructed with usearch -usearch_global and the options ‘-strand plus -id 0.97’. OTU sequences were aligned using ssu-align 0.1.1 [25] and a phylogenetic tree built using FastTree 2.1.8. The entire procedure was run as a Snakemake pipeline [26] and code for the pipeline (version 1.0.1) is available on Github (https://github.com/ucvm/vmmp).

### Diversity analysis

Downstream analysis was done in R 3.3.1 [27] using phyloseq 1.16.2 [28] and vegan 2.4-1 [29]. Mitochondrial and chloroplast sequences were removed as well as the top 20 most abundant OTUs that were present in the negative control samples (Additional file 1: Figure S1). Samples with less than 1000 sequences were also removed from downstream analysis (*n* = 1). Alpha-diversity was measured using Chao1 and Shannon index [30] (using the whole OTU table) [31]. Differences in alpha-diversity between groups was tested using a Mann-Whitney test, controlling the false discovery rate [32] and using a cut-off of *p* < 0.05 for rejecting the null hypothesis of no difference between groups. For β-diversity, only OTUs with a count of 2 or more in at least 10% of the samples were retained for further analysis. Between-sample diversity was evaluated using the Bray-Curtis distance metric on proportionally normalized OTU counts and visualized with non-metric multidimensional scaling (NMDS). In this manuscript, we refer to the OTU counts as abundance and the proportionally normalized counts as relative abundance. The generalized linear model framework as implemented in DESeq2 [33] was used to identify OTUs associated with sample type differences (upper versus lower airways), differences between untreated healthy and disease horses, and the effect of dexamethasone treatment. This approach appropriately controls for over-dispersed data and variable library sizes [34]. A *p*-value cut-off of 0.05 was specified for rejecting the null hypothesis of no difference between groups.

## Results

### Bronchoalveolar lavages results and enrolment

Bronchoalveolar lavages results are shown in Table 1. Seven horses were enrolled in the IAD group based on BAL cytology. Six horses were classified as healthy based on their BAL cytology; three were randomly enrolled in the DEX group and received dexamethasone for 10 days and three were enrolled in the CONTROL group. There was no significant difference in differential cell count for any cell type in the BAL fluid between day 0 and day 11 (Table 1).

**Table 1:** Median (IQR) values for cytologic evaluation of bronchoalveolar fluid obtained before (Day 0) and after 10 days of treatment (Day 11) with intramuscular dexamethasone (0.05 mg/kg SID) for horses with Inflammatory Airway Disease (IAD), and healthy horses (DEX). CONTROL horses were kept in the same environment as both IAD and DEX groups and were sampled at the same time-points, but were given no dexamethasone

Variable	IAD (7 horses)		DEX (3 horses)		CONTROL (3 horses)	
	Day 0	Day 11	Day 0	Day 11	Day 0	Day 11
Neutrophils (%)	10 (6.2 – 11.0)	11 (6.0 - 19.7)	3 (2.0 - 4.75)	3 (1.7 – 3.0)	4 (2.5 – 4.0)	2 (1.7 - 3.2)
Mast cells (%)	8 (5.2 – 11.0)	6.5 (4.2 - 7.2)	2 (2.0 – 2.0)	2 (1.5 - 2.2)	2 (1.7 - 2.7)	2 (1.5 - 2.7)
Eosinophils (%)	0 (0.0 – 0.0)	0 (0.0 – 0.0)	0 (0.0 – 0.0)	0 (0.0 – 0.0)	0 (0.0 - 0.2)	0 (0.0 – 0.0)
Macrophages (%)	49 (45.5 – 53.0)	44 (37.0 - 55.5)	62 (53.5 – 64.0)	69 (66.5 - 74.5)	67 (57.0 - 74.2)	62 (61.5 - 63.5)
Lymphocytes (%)	34.5 (31.7 – 36.0)	31 (23.2 - 42.5)	35 (32.0 - 40.7)	27 (21.0 – 30.0)	25.5 (20.5 - 36.2)	35.5 (31.2 - 35.7)

### Microbiota overview

An average of 13,524 sequences per sample (min: 1219; max: 81,289) were obtained after removal of (i) contaminating OTUs identified in the control samples (*n* = 20 OTUs; Additional file 1: Figure S1), and (ii) samples with less than 1000 sequences (*n* = 1). Using de-novo clustering, 2209 OTUs were identified and 963 OTUs remained after filtering low abundance and rare OTUs.

### Upper and lower respiratory tract microbiota in healthy horses

**Fig. 2:**
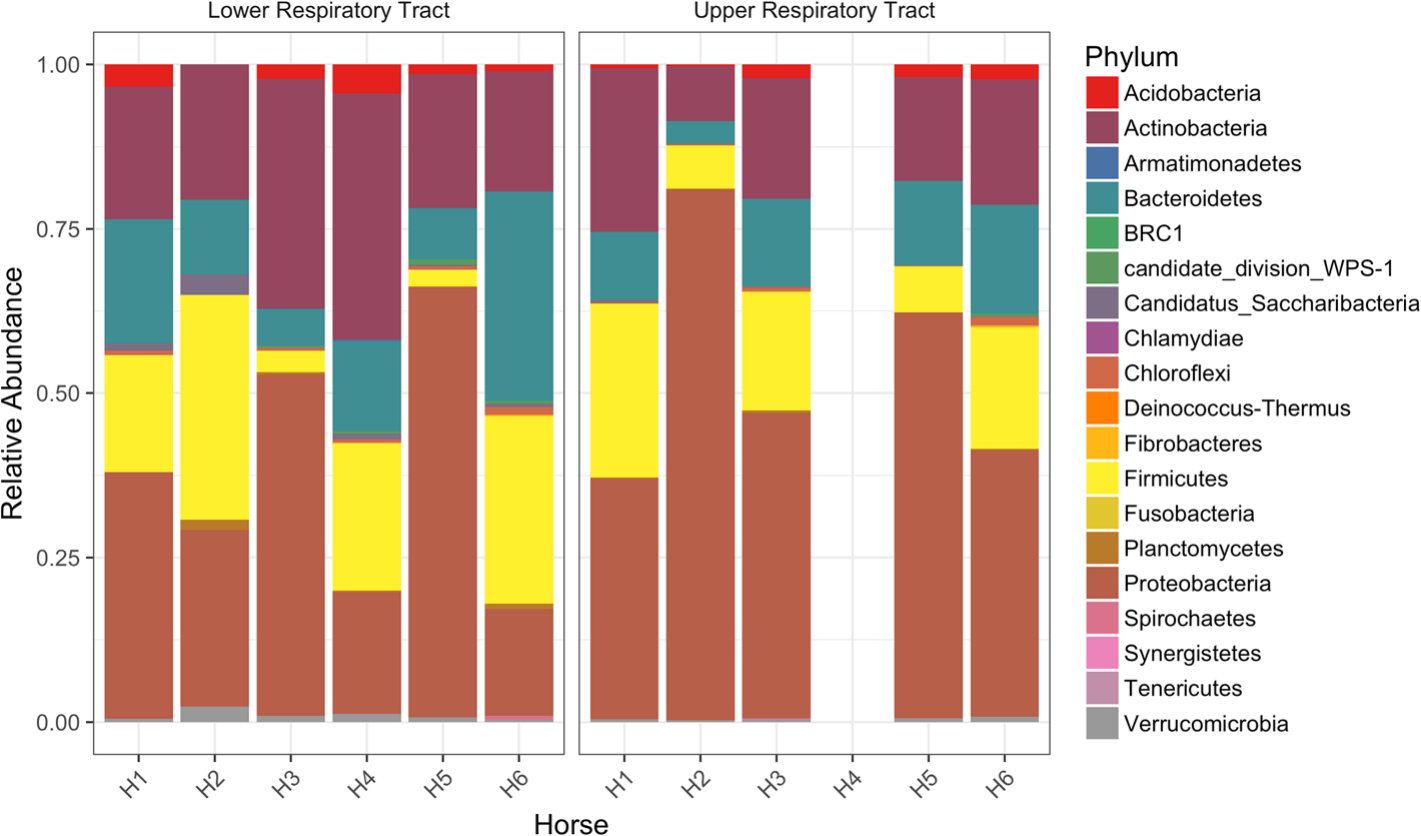
Phyla relative abundance in the upper and lower respiratory tract in the healthy horses (*n* = 6; H1 to H6). Note that the upper respiratory tract sample for horse 4 (H4) had low numbers of sequences after filtering contaminants and was discarded

Nineteen phyla were identified in the respiratory tract of healthy horses at day 0 (*n* = 6), with six phyla showing a relative abundance > 0.1%: Actinobacteria, Bacteroidetes, Chloroflexi, Firmicutes, Proteobacteria and Verrucomicrobia (Table 2, Fig. 2). Four phyla represented 95.54% of the total abundance: Proteobacteria (43.85%), Firmicutes (16.82%), Bacteroidetes (13.24%) and Actinobacteria (21.63%) (Additional file 2: Figure S2). At the genus level, 50 genera had a relative abundance > 0.1% with Sphingomonas and Pantoea being the most abundant (Table 2).

**Table 2:** Relative abundance of the 6 dominant phyla observed in the upper and lower respiratory tract of healthy horses (*n* = 6) at day 0, and relative abundance of genus within each phylum

Phylum (mean relative abundance, %)	Genus (mean relative abundance per phylum, %)
Proteobacteria (43.85%)	Sphingomonas (35.69%)
	Pantoea (26.65%)
	Pseudomonas (14.57%)
	Massilia (5.59%)
	Rhizobium (3.35%)
	Mesorhizobium (2.20%)
	Naxibacter (1.60%)
	Serratia (1.39%)
	Devosia (1.32%)
Actinobacteria (21.63%)	Agrococcus (15.67%)
	Knoellia (12.98%)
	Arthrobacter (12.1%)
	Microbacterium (8.56%)
	Corynebacterium (7.27%)
	Brachybacterium (5.93%)
	Ornithinimicrobium (5.06%)
	Brevibacterium (4.06%)
	Rhodococcus (4.03%)
	Kocuria (3.88%)
	Dietzia (3.71%)
	Rothia (2.5%)
	Clavibacter (1.87%)
	Rathayibacter (1.65%)
	Marmoricola (1.55%)
	Streptomyces (1.29%)
	Nocardioides (1.24%)
Firmicutes (16.82%)	Jeotgalicoccus (30.8%)
	Planomicrobium (19.52%)
	Gemella (16.63%)
	Atopostipes (5.91%)
	Bacillus (4.68%)
	Staphylococcus (3.6%)
	Sporosarcina (2.55%)
	Facklamia (2.23%)
	Trichococcus (1.85%)
	Streptococcus (1.79%)
	Carnobacterium (1.72%)
	Aerococcus (1.23%)
Bacteroidetes (13.24%)	Hymenobacter (42.65%)
	Pedobacter (17.93%)
	Prevotella (17.42%)
	Flavisolibacter (12.19%)
	Gillisia (3.1%)
	Chryseobacterium (2.69%)
	Cloacibacterium (1.66%)
	Ferruginibacter (1.51%)
Verrucomicrobia (0.79%)	Luteolibacter (20.93%)
	Akkermansia (8.79%)
Chloroflexi (0.51%)	Sphaerobacter (97.84%)
	Litorilinea (2.16%)

**Fig. 3:**
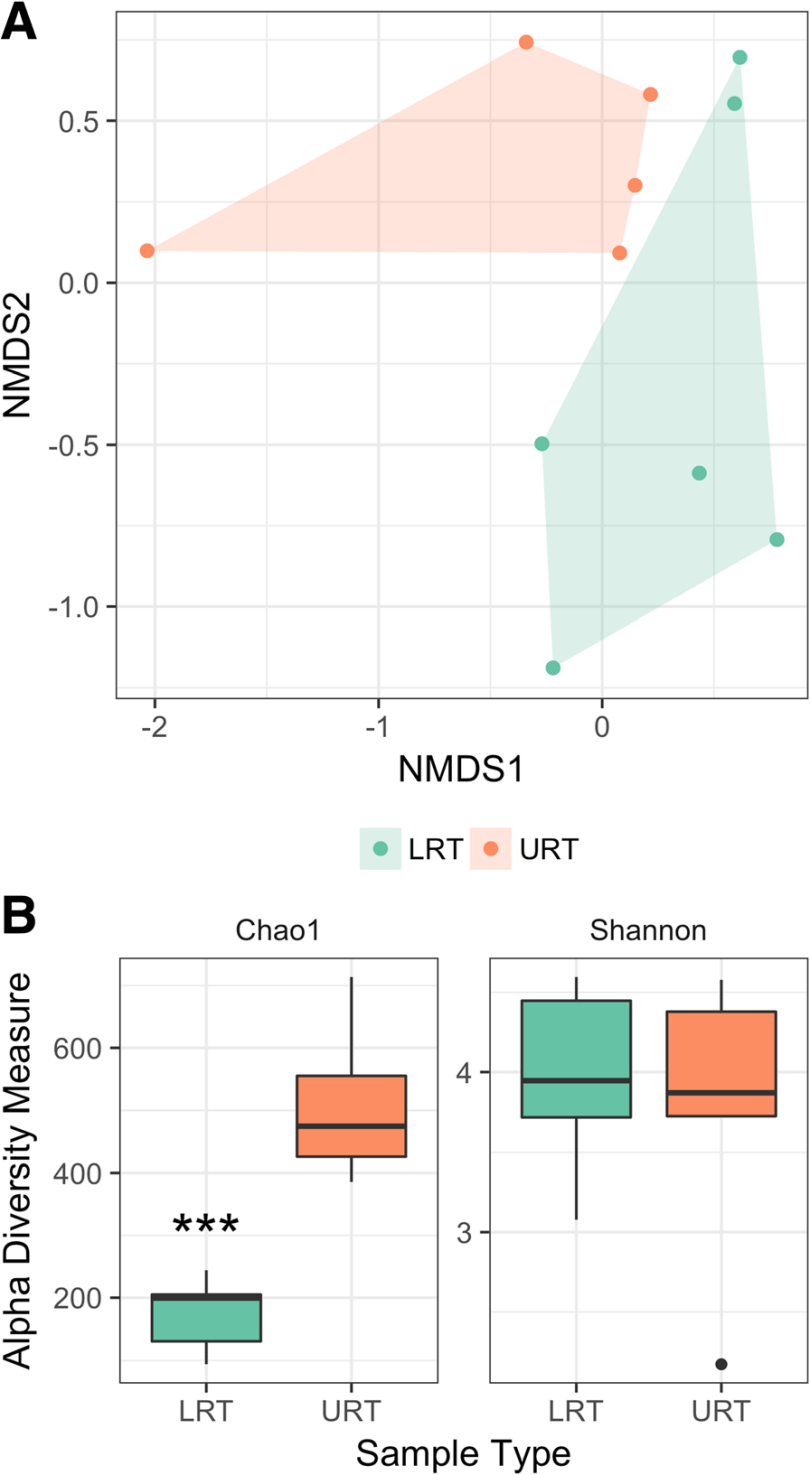
**a**: Nonmetric multidimensional scaling (NMDS) ordination with Bray-Curtis distance of the upper respiratory tract (URT) and lower respiratory tract (LRT) in healthy horses (*n* = 6). **b**: Alpha diversity measures (Chao1 and Shannon) in both upper and lower respiratory tract samples. *** significant decrease in the richness of the local bacterial community in the lower respiratory tract (LRT) compared to the upper respiratory tract (URT) (*p* = 0.0043, Wilcoxon test) in healthy horses (*n* = 6)

Nonmetric multidimensional scaling (NMDS) ordination with Bray-Curtis distance showed differences in bacterial communities between the upper and lower respiratory tract of healthy horses (Fig. 3a). Testing for a difference between the upper and lower respiratory tract microbiota revealed a change in the relative abundance of 2 OTUs, with Moraxella increased in the upper respiratory tract, and Cupriavidus increased in the lower respiratory tract (Additional file 3: Figure S3). There was also a significant decrease in richness at the lower respiratory tract level based on Chao1 (*p* = 0.0043, Wilcoxon test) (Fig. 3b). However, Shannon index did not differ between the upper and lower respiratory tract (*p* = 0.93, Wilcoxon test) (Fig. 3b), indicating that while there was an overall decrease in species richness, evenness remained unchanged.

### Differences between healthy and IAD horses

**Fig. 4:**
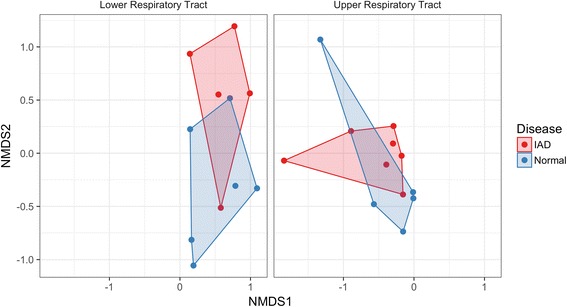
Nonmetric multidimensional scaling (NMDS) ordination with Bray-Curtis distance of the differences in bacterial communities at the upper respiratory tract and lower respiratory tract level between disease status (Inflammatory Airway Disease [IAD], *n* = 7; Normal, *n* = 6)

**Fig. 5:**
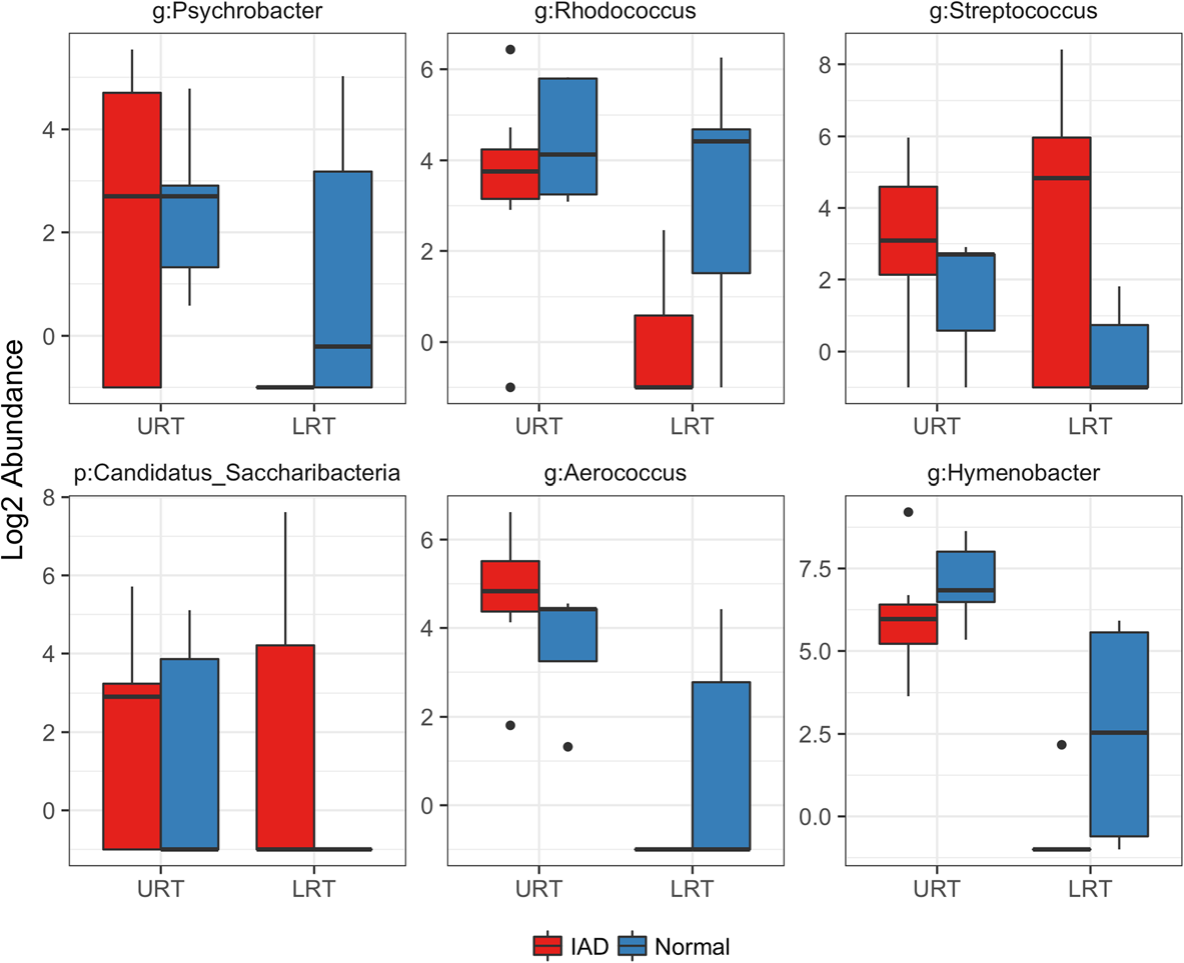
Abundance of 6 OTUs that are statistically different in horses with Inflammatory Airway Disease (IAD) (*n* = 7; *red bars*) compared to healthy controls (*n* = 6; *blue bars*) in the Lower Respiratory Tract (LRT) from samples obtained on day 0 (prior to dexamethasone treatment). No differentially abundant OTUs were identified between disease status in the upper respiratory tract (URT). Each panel shows the abundance for an individual OTU and is labelled with the taxa and taxa rank (p: Phylum or g: Genus) that was assigned to it

Differences in bacterial communities at the upper and lower respiratory tract level between disease status were visualized using NMDS (Fig. 4). Between healthy and IAD horses, 6 OTUs (Fig. 5) differed in the lower airways based on the generalized linear model. In horses with IAD, relative abundance of *Streptococcus* and an OTU assigned to the phylum Candidatus_Saccharibacteria were increased, whereas relative abundance of *Psychrobacter*, *Rhodococcus, Aerococcus* and *Hymenobacter spp.* were decreased (Fig. 5). No differentially abundant OTUs were identified between disease status in the upper respiratory tract.

### Effects of dexamethasone on the upper and lower respiratory tract microbiota

**Fig. 6:**
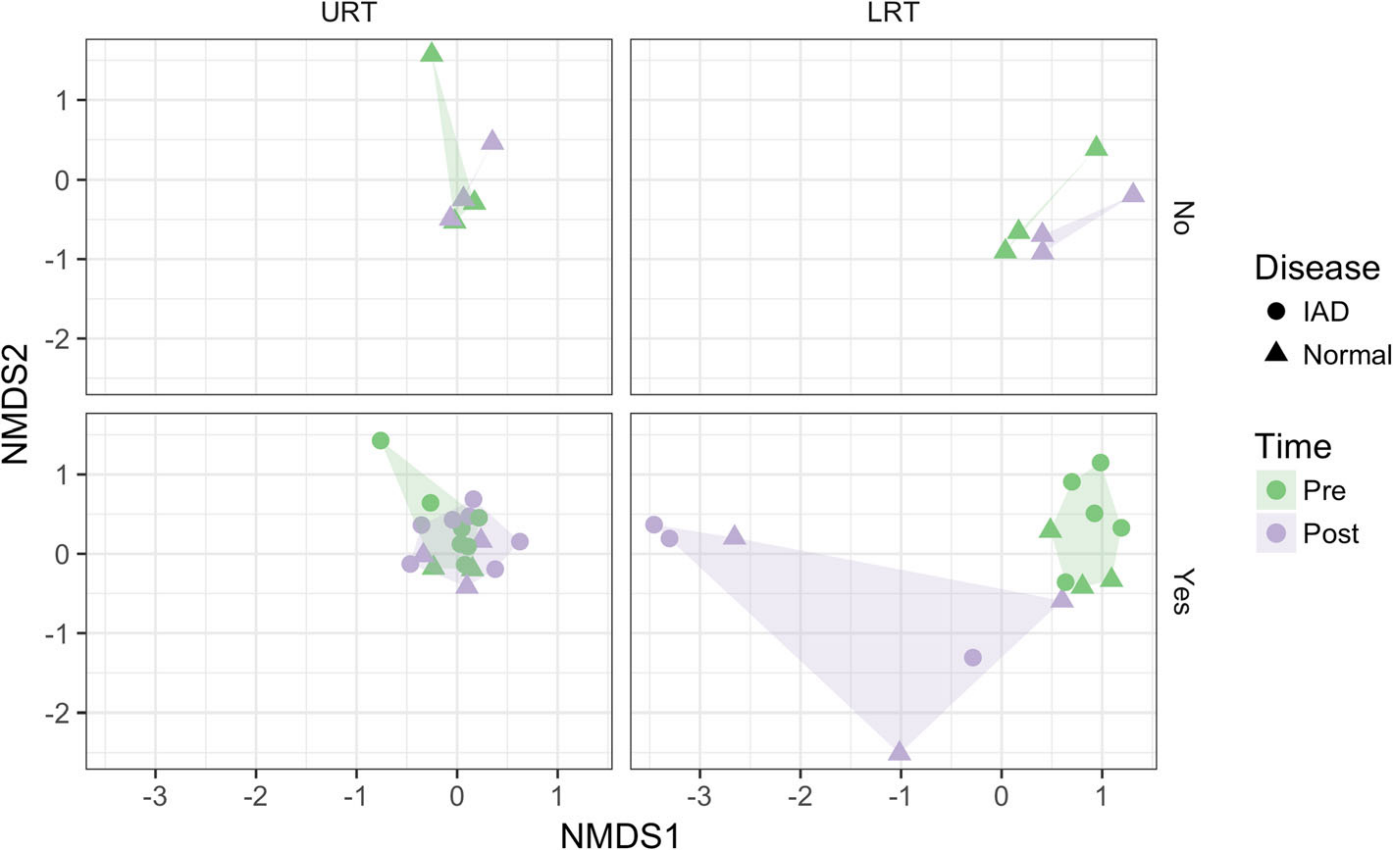
Nonmetric multidimensional scaling (NMDS) ordination with Bray-Curtis distance of the dexamethasone treatment effect on the lower respiratory tract microbiota of both healthy (Normal; *n* = 6; Triangles) and Inflammatory Airway Disease (IAD; *n* = 7; Circles) horses (“No”: indicates no treatment: *n* = 3 Normal horses. “Yes”: indicates dexamethasone treatment for 10 days: *n* = 3 Normal horses and *n* = 7 IAD horses). The *horizontal axis* is the Upper Respiratory Tract (URT) and Lower Respiratory Tract (LRT), and the *vertical axis* is dexamethasone treatment (CONTROL group did not receive dexamethasone). Pre and Post indicate day 0 and day 11 sampling time-points, respectively

**Fig. 7:**
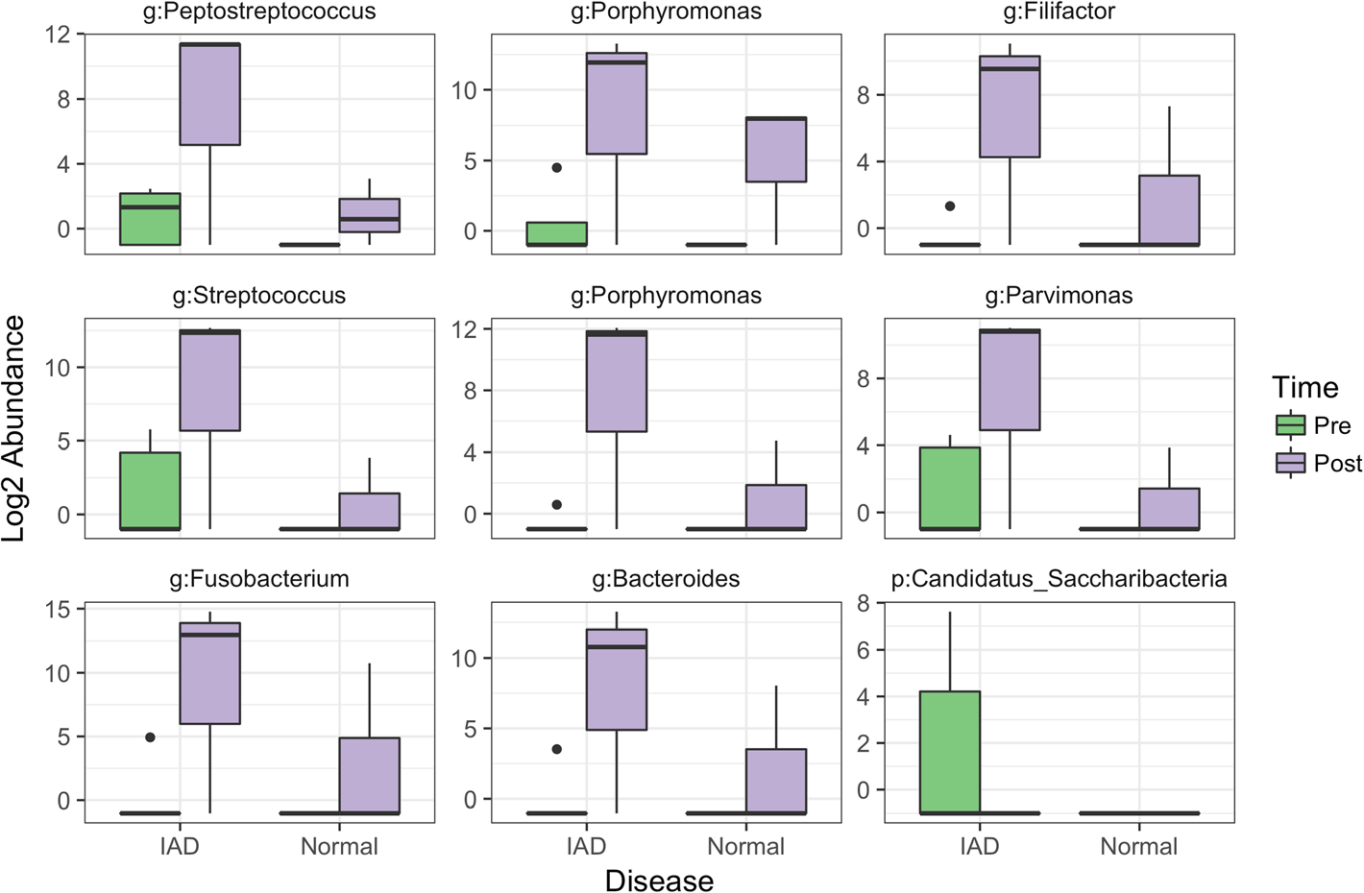
Overlapping treatment effect of dexamethasone administration for 10 days (Pre: Day 0; *green bars*, and Post Day 11; *purple bars*) in healthy horses (Normal; *n* = 3) and horses with Inflammatory Airway Disease (IAD; *n* = 7). Each panel shows the abundance for an individual OTU and is labelled with the taxa and taxa rank (p: Phylum or g: Genus) that was assigned to it

Dexamethasone had an effect on the lower respiratory tract microbiota of both healthy and IAD horses (Fig. 6); the treatment effect was not different between disease status. After 10 days of dexamethasone administration, the lower respiratory tract of both healthy and IAD horses experienced a significant change in the abundance of 11 OTUs (Additional file 4: Figure S4), with 9 OTUs responding similarly between disease status (Fig. 7). *Peptostreptococcus, Porphyromonas, Filifactor, Streptococcus, Porphyromonas, Parvimonas, Fusobacterium* and *Bacteroides* spp. increased from day 0 to day 11 (pre vs post treatment), whereas Candidatus_Saccharibacteria OTU decreased. There was evidence that dexamethasone treatment also decreased evenness in the lower airways of both healthy and IAD horses, however, this decrease was not statistically significant based on our pre-set level of significance (Additional file 5: Figure S5). No treatment effect was observed on the upper respiratory tract microbiota (Fig. 6).

No differentially abundant OTUs were detected between time points (d 0 and d 11) for the CONTROL group (i.e. healthy horses not treated with dexamethasone; data not shown) indicating that time alone did not significantly influence the airways microbiota.

## Discussion

This study investigated for the first time the differences in airway community profiles between healthy horses and horses with mild equine asthma (IAD), and the evolution of these communities after dexamethasone treatment. Using high throughput sequencing, we showed a difference in the lower respiratory tract between healthy and IAD horses, with 6 OTUs having different abundances. However, no differences were observed between disease status at the upper respiratory tract level. Dexamethasone induced a distinct shift in the community structure of the lower respiratory tract in both healthy horses and those with IAD, with a significant change in the abundance of 11 OTUs, with 9 OTUs responding similarly to treatment. A difference in community structure was also observed between the upper and lower respiratory tract of healthy horses.

Strengths of our study design include strict attention to controls, with the inclusion of negative controls at sample collection, during extraction, and throughout sequencing. This resulted in the removal of 20 potential contaminants from analysis. Previous reports warned that contamination is a critically important issue in sequence-based microbiome analysis, particularly when samples contain a low biomass [35, 36]. Another strength of our study was the technique used to collect the lower respiratory tract samples, the percutaneous transtracheal method, which is superior at avoiding contamination than using a sheathed catheter through an endoscope [18, 37]. The OTUs filtering protocol used was also stringent, which limited the impact of contamination on results presented. Finally, all horses resided on the same property and paddock, which controlled for the potential confounding variables such as location, environmental management, and husbandry procedures. A limitation of this study is its small sample size (*n* = 13), which could have impaired our ability to detect differences in community profiles and/or OTUs between treatment groups (type 2 error). While we did not observe any apparent outliers in the studied population, the results presented in this manuscript must be interpreted with consideration of the small sample size; results might not generalise well to other equine communities.

The differences observed between healthy and IAD horses at the lower respiratory tract level concurred with the previous report indicating that bacteria could play a role in the pathogenesis of mild equine asthma [9–11]. However, in the present study, only the abundance of *Streptococcus* spp. was increased in horses with IAD and we did not observe a significant increase in *Actinobacillus* spp., *Acinetobacter* spp. and *Mycoplasma* spp. as reported previously [9]. Interestingly, the presence of *Streptococcus (S. zooepidemicus and S*. *pneumonia*) in tracheal washes were previously associated with lower airway inflammation in a study conducted on 278 Thoroughbred racehorses [38]. Furthermore, a study on human asthma also reported that the genera with the highest relative abundance in bronchial biopsy samples was *Streptococcus* [39]. Based on this finding, it seems that further research is warranted on the role of *Streptococcus* on asthma pathogenesis in horses.

As expected, dexamethasone had a significant treatment effect on the microbiota of the lower respiratory tract in horses. After dexamethasone treatment, numerous OTUs increased in abundance, including *Streptococcus spp.*, while Candidatus_Saccaribacteria OTU decreased in abundance*.* This overgrowth of certain bacteria in the lower respiratory tract could be secondary to the immunomodulation induced by the dexamethasone treatment, with the reduction in Candidatus_Saccaribacteria OTU providing supportive evidence that dexamethasone treatment also decreased evenness in the lower airways of both healthy and IAD horses. In humans, macrolide antibiotic administration has been used in addition to bronchodilators and corticosteroids to improve lung function in asthmatic patients [40]. Perhaps bacterial overgrowth in IAD horses treated with dexamethasone should be controlled to improve treatment success, especially in cases with a poor response to corticosteroids therapy.

The treatment effect of dexamethasone was not different between healthy horses and those with IAD, indicating either (i) that inflammation present in the lower respiratory tract does not influence the immunosuppressive effects of dexamethasone, (ii) that dexamethasone has a stronger effect than the disease on the microbiota, or (iii) that we did not have enough power to detect a difference. We did not include a control group of untreated horses with IAD for ethical reasons as they were privately owned horses; however, there were no OTUs that differed in abundance over the course of the trial in the CONTROL group, thus indicating that the difference observed in the groups treated with dexamethasone was due to the treatment. While dexamethasone administration has an effect on the lower respiratory tract microbiota, it is interesting to note its lack of effect on BAL cytology in the present study. This finding is consistent with other equine asthma studies, performed on horses with both mild and severe asthma, where an improvement in pulmonary function and clinical signs was not associated with a concurrent decrease in inflammatory cells in BAL [15, 41, 42].

This study demonstrates that the equine lung is not sterile, with the lower respiratory tract possessing a unique microbiota. However, only 2 OTUs differed between upper and lower airways, indicating that the majority of OTUs were overlapping. Interestingly, in healthy humans, the nasal microbiota contributed very little to the composition of the local bacterial communities in the lung, however, there was an overlap with the communities observed in the mouth [43]. That numerous OTUs are shared by the upper and lower tract in the present study can be explained by the fact that horses are obligate nasal breathers and have complete separation of the nasopharynx and oral cavity, except when swallowing, due to an elongated soft palate.

## Conclusions

The respiratory microbiome of horses is diverse, but dominated by four phyla, *Proteobacteria*, *Firmicutes*, *Bacteroidetes* and *Actinobacteria*. There was a clear separation between the bacterial community in the lower respiratory tract of healthy and IAD horses, with 6 OTUs in the tracheal community having different abundance with disease status, including an increase in *Streptococcus* in horses with IAD. Based on this finding, further research is warranted on the role of *Streptococcus* on IAD pathogenesis in horses. Treatment with dexamethasone has a significant treatment effect on the lower respiratory tract microbiome in all horses, with numerous OTUs increasing in abundance, including *Streptococcus spp.* Perhaps control of bacterial overgrowth in IAD horses treated with dexamethasone could be part of the treatment strategy.

## Additional files

Additional file 1: Figure S1. Abundance of contaminating OTUs identified in the blank samples (*n* = 20). Blank negative controls did not include water (kit only). (TIFF 21097 kb)

Additional file 2: Figure S2. Top 10 genera (by relative abundance) in each of the major phyla identified in the upper and lower respiratory tract of 6 healthy horses (H1 to H6). (TIFF 35863 kb)

Additional file 3: Figure S3. Abundance of 2 OTUs (labelled with genus) that differed between the upper respiratory tract (URT) and lower respiratory tract (LRT) of healthy horses (*n* = 6). (TIFF 14065 kb)

Additional file 4: Figure S4. Dexamethasone treatment (10 days) effect (Pre: day 0; green bars and Post: day 11; purple bars) in the lower respiratory tract of both healthy (*n* = 6) and Inflammatory Airway Disease (IAD, *n* = 7) horses. Each panel shows the abundance for an individual OTU and is labelled with the taxa and taxa rank (p: Phylum or g: Genus) that was assigned to it. (TIFF 42192 kb)

Additional file 5: Figure S5. Alpha diversity measures (Chao1 and Shannon) of dexamethasone treatment (10 days) effect (Pre: day 0; green bars and Post: day 11; purple bars) in both upper respiratory tract and lower respiratory tract samples. There was no significant decrease in the evenness in the lower airways of both healthy (Normal) and Inflammatory Airway Disease (IAD) horses after *p*-value adjustment for multiple comparisons (*p* = 0.071, Wilcoxon test). (TIFF 8440 kb)

## Abbreviations

BAL: Bronchoalveolar Lavage
CONTROL: Healthy control group (no dexamethasone) (*n* = 3)
DEX: Healthy dexamethasone group (*n* = 3)
IAD: Inflammatory Airway Disease (mild equine asthma)
LRT: Lower Respiratory Tract
NMDS: Nonmetric Multidimensional Scaling
NPS: Nasopharyngeal Swab
OTU: Operational Taxonomic Unit
TTW: Transtracheal Wash
URT: Upper Respiratory Tract
