# Supplementary figures and images for: Upper and lower respiratory tract microbiota in horses: bacterial communities associated with health and mild asthma (inflammatory airway disease) and effects of dexamethasone
Source: BMC Microbiol. 2017 Aug 23;17:184. doi: 10.1186/s12866-017-1092-5 (PMC5569571; doi:10.1186/s12866-017-1092-5)

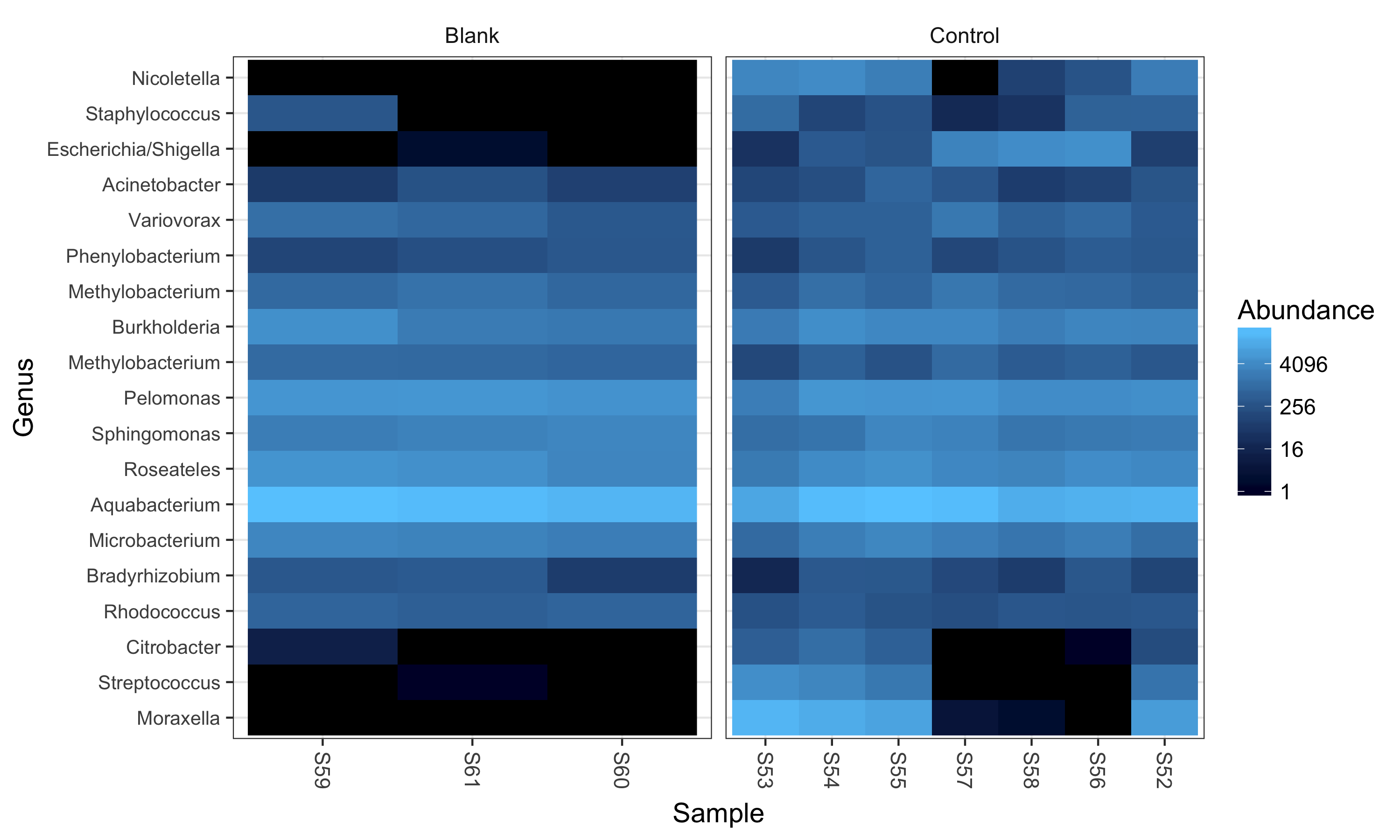

Supplement: Supplementary file 1 — Abundance of contaminating OTUs identified in the blank samples (n = 20). Blank negative controls did not include water (kit only). (TIFF 21097 kb) [file 12866_2017_1092_MOESM1_ESM.tiff]

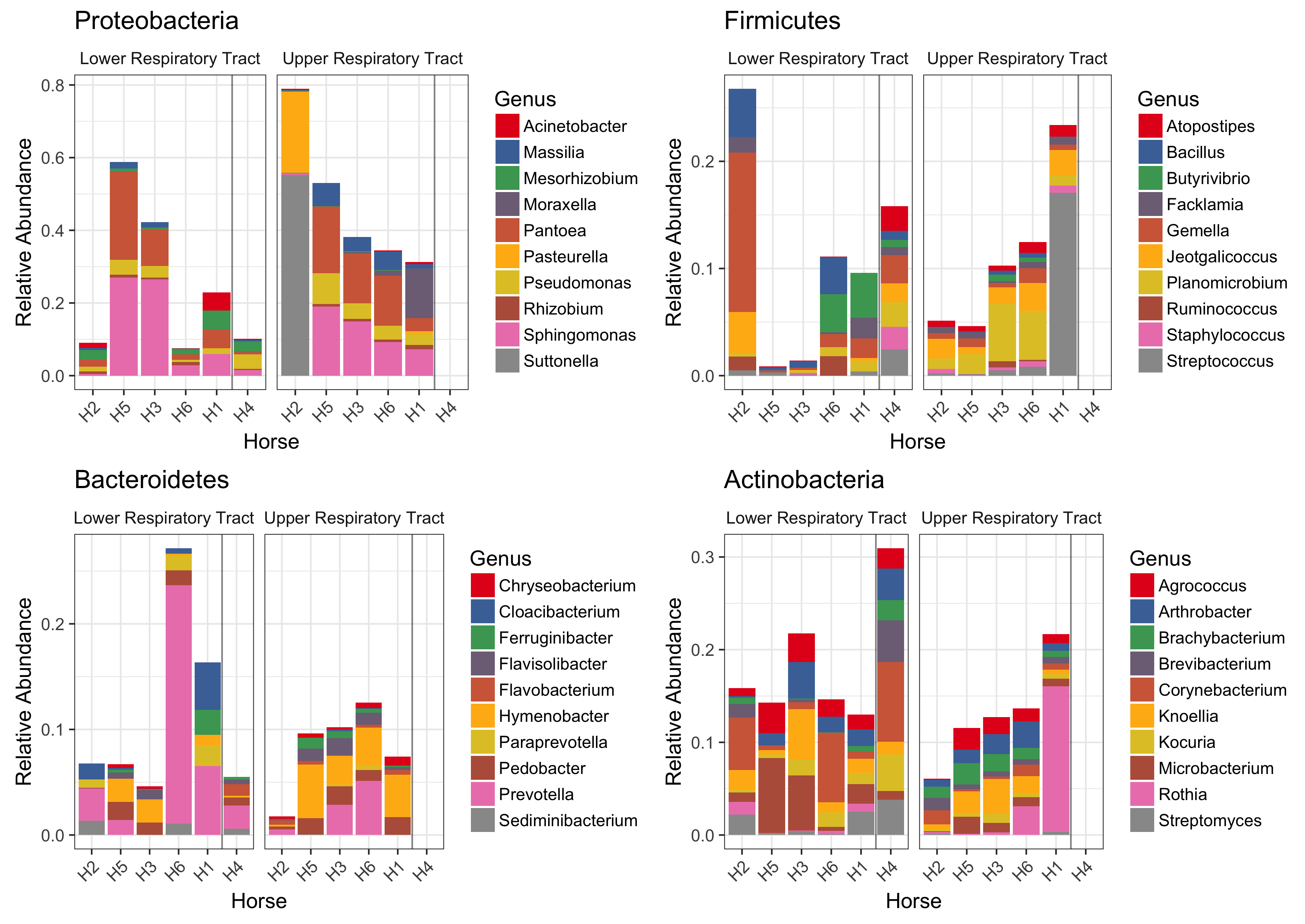

Supplement: Supplementary file 2 — Top 10 genera (by relative abundance) in each of the major phyla identified in the upper and lower respiratory tract of 6 healthy horses (H1 to H6). (TIFF 35863 kb) [file 12866_2017_1092_MOESM2_ESM.tiff]

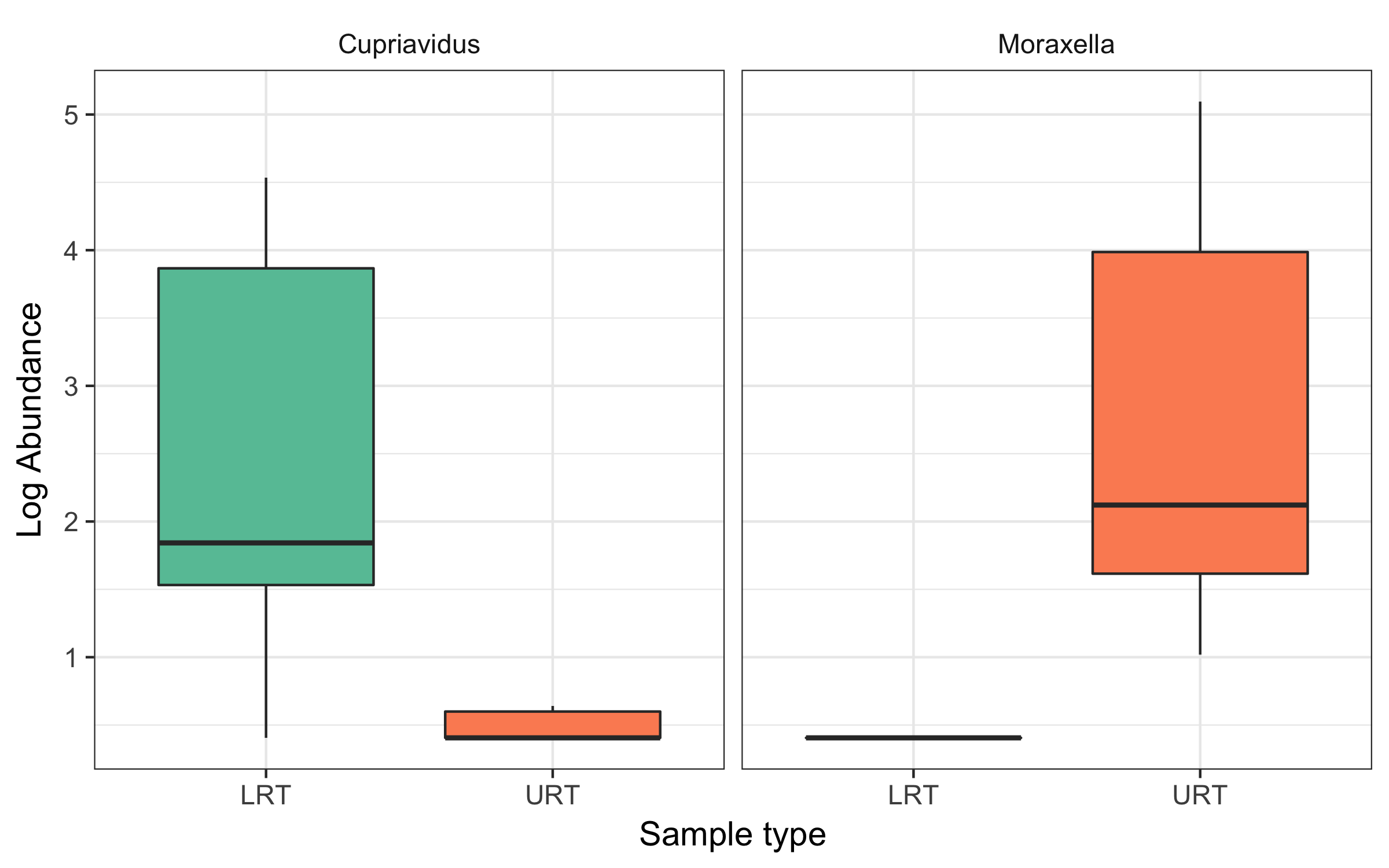

Supplement: Supplementary file 3 — Abundance of 2 OTUs (labelled with genus) that differed between the upper respiratory tract (URT) and lower respiratory tract (LRT) of healthy horses (n = 6). (TIFF 14065 kb) [file 12866_2017_1092_MOESM3_ESM.tiff]

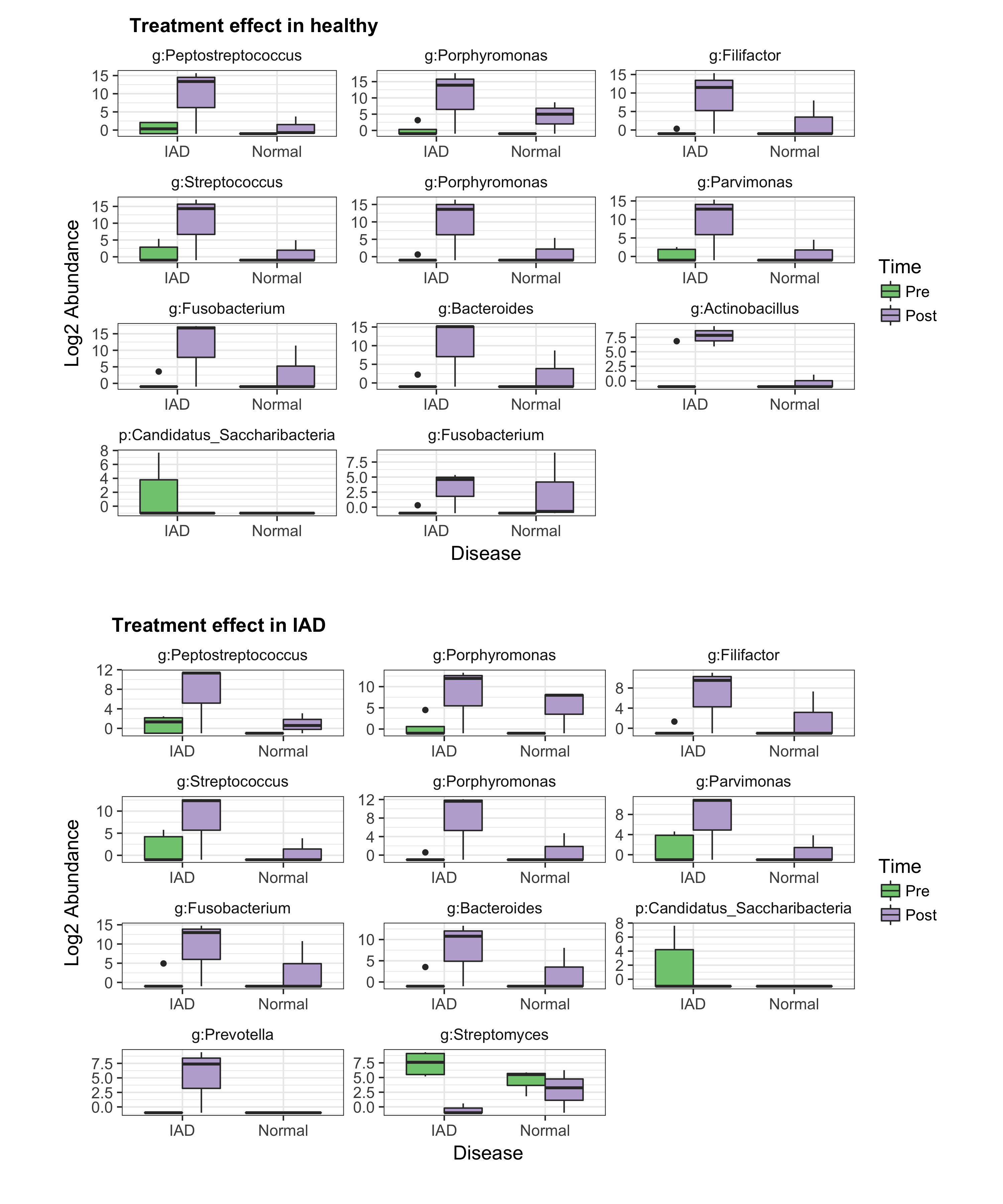

Supplement: Supplementary file 4 — Dexamethasone treatment (10 days) effect (Pre: day 0; green bars and Post: day 11; purple bars) in the lower respiratory tract of both healthy (n = 6) and Inflammatory Airway Disease (IAD, n = 7) horses. Each panel shows the abundance for an individual OTU and is labelled with the taxa and taxa rank (p: Phylum or g: Genus) that was assigned to it. (TIFF 42192 kb) [file 12866_2017_1092_MOESM4_ESM.tiff]

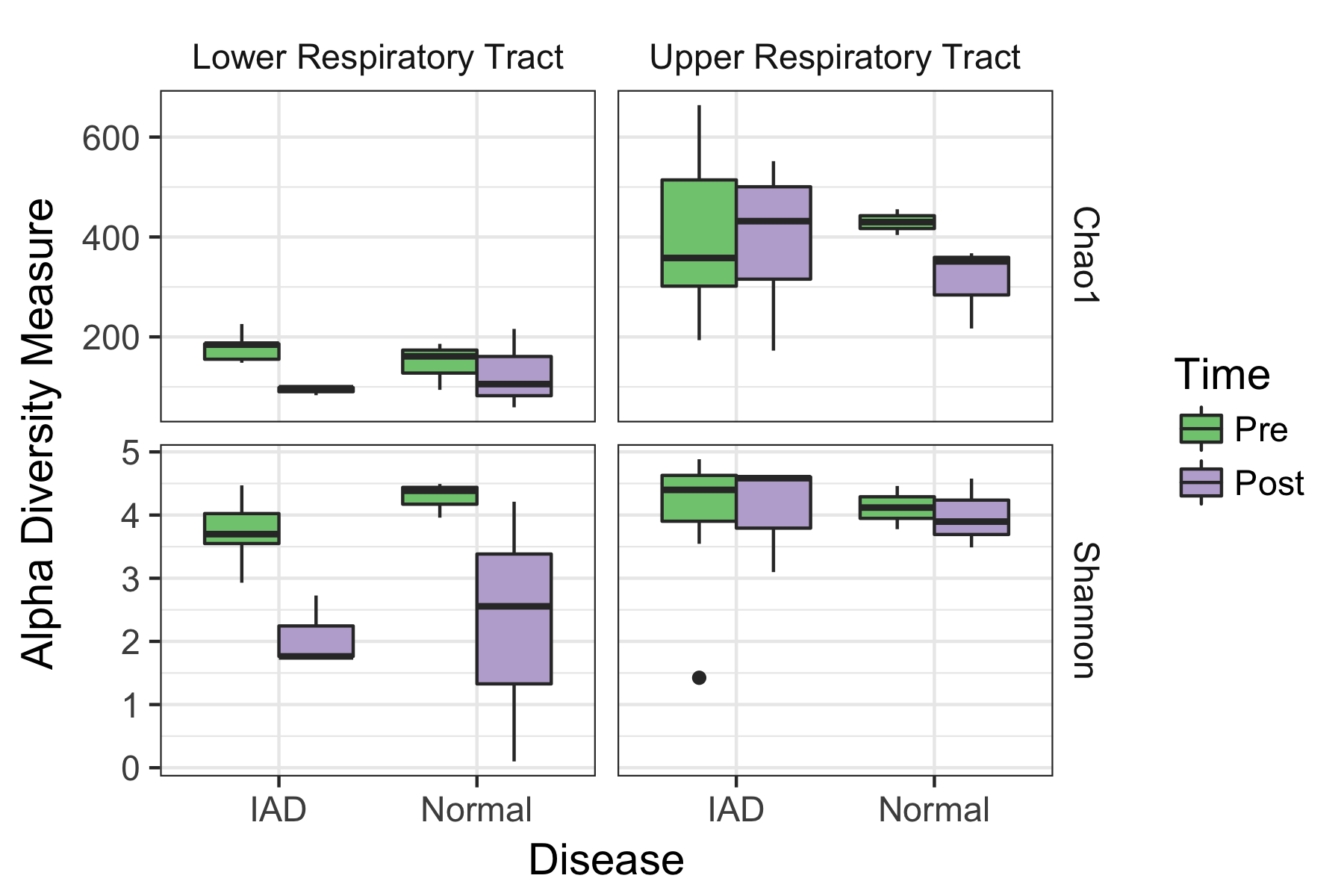

Supplement: Supplementary file 5 — Alpha diversity measures (Chao1 and Shannon) of dexamethasone treatment (10 days) effect (Pre: day 0; green bars and Post: day 11; purple bars) in both upper respiratory tract and lower respiratory tract samples. There was no significant decrease in the evenness in the lower airways of both healthy (Normal) and Inflammatory Airway Disease (IAD) horses after p-value adjustment for multiple comparisons (p = 0.071, Wilcoxon test). (TIFF 8440 kb) [file 12866_2017_1092_MOESM5_ESM.tiff]
